# Bio-Based Polymers with Potential Antimicrobial Activity from Vanillin Methacrylate via ARGET-ATRP

**DOI:** 10.3390/polym18091023

**Published:** 2026-04-23

**Authors:** Eddy Marelli, Maristella Mastore, Maurizio F. Brivio, Francesco Della Monica, Lorella Izzo, Orlando Santoro

**Affiliations:** 1 Laboratory of Polymer Chemistry and Sustainable Catalysis, Department of Biotechnology and Life Sciences (DBSV), University of Insubria, 21100 Varese, Italy; emarelli1@uninsubria.it (E.M.); f.dellamonica@uninsubria.it (F.D.M.); lorella.izzo@uninsubria.it (L.I.); 2 Laboratory of Applied Entomology and Parasitology, Department of Theoretical and Applied Sciences (DiSTA), University of Insubria, 21100 Varese, Italy; maristella.mastore@uninsubria.it (M.M.); maurizio.brivio@uninsubria.it (M.F.B.)

**Keywords:** vanillin methacrylate, ARGET-ATRP, bio-based polymers, biomass valorization, antimicrobial polymers

## Abstract

The Activators Regenerated by Electron Transfer Atom Transfer Radical Polymerization (ARGET-ATRP) of vanillin methacrylate (**VMA**), a bio-based methacrylic monomer derived from vanillin, was systematically studied for the first time. The reaction conditions were optimized aiming at achieving good monomer conversions while preserving the antimicrobial aldehyde functionality. Bipyridine-based catalysts showed limited effectiveness, whereas polydentate aliphatic amines displayed higher activity. Kinetic studies showed linear profiles during the early stages of the polymerization before reaching a conversion plateau accountable to the depletion of the reducing agent, as confirmed by reactivation experiments. The resulting polymer (**PVMA**) exhibited a glass transition temperature comparable to that of poly(styrene), emerging as a potential bio-derived alternative to fossil-based thermoplastic materials. Furthermore, preliminary in vitro tests demonstrated that **PVMA** has potential antimicrobial activity against both *Escherichia coli* (Gram-negative) and *Bacillus subtilis* (Gram-positive).

## 1. Introduction

Over the past decades, the forecasted complete depletion of fossil sources within the next century, geopolitical instability generating high price fluctuations of oil-derived products, along with the need for more sustainable, environmentally friendly materials, motivated researchers to devote efforts towards the development of synthetic polymers that, while retaining the properties of their ubiquitous fossil-based congeners, are obtained from bio-derived monomers [1,2,3,4,5,6]. Indeed, thermoplastics such as bio-polyethylene (Bio-PE), bio-polypropylene (Bio-PP) and bio-poly(ethylene terephthalate) (Bio-PET) already find day-by-day applications [7]. Aside from employing *conventional* monomers not originated from petroleum, the use of alternative building blocks obtainable from waste/biomass has been gaining increasing attention, allowing for the production of a wide range of polymers spanning from thermoplastics to elastomers [8,9,10,11,12,13,14,15]. Many of these *alternative* polymers are based on naturally occurring phenols, which possess functional groups that can serve as post-functionalization sites enabling the design of materials with tailored features [16,17,18]. On the other hand, such functionalities can be preserved, conferring intrinsic properties such as antimicrobial/antioxidant activity [19]. Similarly, vanillin (**VA**) represents a particularly promising bio-based building block, as it can be obtained from lignin depolymerization as well as the biodegradation of poly(ethylene terephthalate) (PET) [20,21] and, owing to the presence of a rigid aromatic ring, its corresponding polymers would exhibit high glass transition temperatures (*T*_g_ > room temperature), comparable to that of oil-derived thermoplastics like poly(styrene) (PS). In addition, the aldehydic functionalities would confer to the material inherent antimicrobial activity [22,23]. **VA** has been extensively employed in combination with other molecules to produce a variety of bio-based materials, including epoxy polymers, phenolic resins, polyesters and polyacetals [24] and its (meth)acrylated derivatives have been used as monomers in free radical polymerization processes [25,26,27]. Nevertheless, preserving the aldehyde function under such reaction conditions often results challenging due to the propensity of such a function to radical harvesting [28,29]. Although controlled radical polymerization approaches, such as reversible addition–fragmentation chain transfer (RAFT) and reverse iodine transfer polymerization (RITP), have been explored for this class of monomers [30,31,32], no systematic studies on the activators regenerated by electron transfer atom transfer radical polymerization (ARGET-ATRP) of vanillin methacrylate (**VMA**) are found to date [33,34]. While RAFT polymerization provides broad monomer versatility, enabling the polymerization of a wide range of monomers and functional groups without significantly inhibiting the reaction, a major limitation lies in the demanding synthesis of specialized chain transfer agents (CTAs) [35]. Conversely, RITP shows limited control over monomers that generate tertiary radicals, such as methyl methacrylate, mainly due to competing side reactions [36] and, in the specific case of VMA, low *M*_n_ were targeted. In this context, ARGET-ATRP emerges as a particularly attractive controlled radical polymerization technique for methacrylic monomers, as it enables precise control over molar mass and dispersity while operating under significantly reduced catalyst concentrations and relatively mild reaction conditions [37].

In this contribution, we report on the unprecedented investigation of **VMA** via ARGET-ATRP, a sustainable polymerization technique, specifically aiming at preserving the aldehyde functional group. The influence of different catalytic systems and the polymerization kinetics are discussed, together with the structural and thermal characterization of the obtained materials. In addition, since a limited number of newly identified antimicrobial compounds advance to clinical development, a process that generally requires extensive in vitro and subsequently in vivo testing, we conducted preliminary in vitro assays to assess the potential application of VMA as a polymer with antimicrobial activity.

## 2. Materials and Methods

### 2.1. Materials

All manipulations involving air-sensitive compounds were carried out under a nitrogen atmosphere using Schlenk techniques. Vanillin (**VA**, >98.0%) and methacrylic anhydride (**MAh**, >97.0%) were purchased from TCI Europe N.V. (Zwijndrecht, Belgium) and used as received. CuCl_2_ (99%), CuBr_2_ (99%), 2-2′-bypyridine (**BiPy**, >99.0%), 4-4′-dimethoxy-2-2′-bipyridine (**DMeOBiPy**, 97%) N-N-N′-N″-N″-pentamethyldiethylenetriamine (**PMDETA**, 99%), 1,1,4,7,10,10-hexamethyltriethylenetetramine (**HMTETA**, 97%), tris[2-(dimethylamino)ethyl]amine (**Me6TREN**, 97%), ethyl α-bromoisobutyrate (**EBiB**, 98%) and tin (II) 2–ethylhexanoate (**Sn(EH)_2_**, 92.5–100%) were purchased from Merk KG (Darmstadt, Germany) and used as received. Vanillin methacrylate (**VMA**) was synthesized by previously reported procedures [24]. Anisole (99.0%) was dried over CaCl_2_ overnight before use. Unless stated otherwise, all solvents (anisole, dimethyl sulfoxide (DMSO), dichloromethane (DCM), 2-propanol, petroleum ether 40–60 and ethyl acetate) were purchased from Carlo Erba Reagents S.r.l. (Milan, Italy) and used as received.

For antimicrobial activity evaluation, all reagents were obtained from Sigma Chemicals (St. Louis, MO, USA) and Merck Millipore Ltd. (Tullagreen, Cork, Ireland). Laboratory instruments were supplied by Bio-Rad Laboratories (Detroit, MI, USA), Euroclone S.p.A. (Milan, Italy), Olympus (Segrate, Italy), and Optika S.r.l. (Ponteranica, Italy). Centrifugation steps were performed using a SIGMA 1–14 microcentrifuge (SciQuip Ltd., Newtown, UK) and an Eppendorf 5804 centrifuge (Eppendorf, Hamburg, Germany). Spectrophotometric measurements were conducted with a Jasco V-560 spectrophotometer (Jasco, Easton, MD, USA). All materials, buffers, and solutions were sterilized either by autoclaving or by filtration through 0.22 µm Minisart filters (Sartorius, Göttingen, Germany).

### 2.2. Methods

Polymer samples were dried in a Memmert VO29 vacuum oven operating at 30 °C and 5 mbar.

Nuclear Magnetic Resonance (NMR) spectra were recorded on a Bruker AV400 instrument (Bruker Corporation, Billerica, MA, USA) operating at 400 MHz in the Fourier Transform mode and at 293 K. The samples (20 mg) were dissolved in 0.5 mL of CDCl_3_. Tetramethylsilane (TMS) was used as an internal chemical shift reference.

Attenuated Total Reflectance—Fourier transform Infra-Red (ATR-FTIR) spectra were recorded on a Cary 630 FTIR spectrometer (Agilent Technologies, Cernusco sul Naviglio, Italy) at room temperature with 16 scans and a resolution of 4 cm^−1^.

Number Average Molecular Weight (*M*_n_) and polydispersity (*Đ*) values were determined by Size Exclusion Chromatography (SEC) analyses against poly(styrene) standards performed on a modified Jasco HPLC system equipped with a Shodex KF-804L column (Showa Denko, Tokyo, Japan), operating at 30 °C in THF with a flow rate of 1 mL/min.

Wettability measurements were performed on an Ossila Contact Angle Goniometer (Ossila Ltd., Sheffield, UK). The reported Contac Angle (CA) values are an average of at least 3 measurements.

Thermal characterization was performed by Differential Scanning Calorimetry (DSC) using a Stare system DSC 3 (Mettler Toledo, Milan, Italy). DSC samples were heated from 25 to 150 °C, cooled down to 0 °C and heated again to 150 °C at a rate of 10 °C/min, using a 50 mL/min nitrogen flow rate. The glass transition temperatures (*T*_g_) were determined from the second heating ramp. The thermal degradation properties were investigated with a Stare system TGA 2 (Mettler Toledo, Milan, Italy) by heating the sample from 25 to 600 °C at a rate of 10 °C/min in a nitrogen atmosphere.

SEM-EDX analyses were performed on an Environmental Scanning Electron Microscope FEI ESEM-FEG XL30 (Philips, Milan, Italy) working in the high vacuum mode and with an acceleration voltage equal to 20.0 kV.

### 2.3. Synthesis of Vanillin Methacrylate (VMA)

According to reference [24], to a 100 mL round-bottomed Schlenk flask equipped with a magnetic stir bar, a catalytic amount of DMAP (0.194 g, 1.6 mmol, 2 mol%) was added to vanillin (**VA**, 10 g, 65.7 mmol, 1 equiv.). The reaction vessel was sealed and purged with nitrogen gas to remove moisture and oxygen before adding methacrylic anhydride (**MAh**, 9.74 mL, 1.01 equiv.). The reaction mixture was stirred at room temperature at 500 rpm for 15 min and then heated to 45 °C for 48 h. The reaction mixture was taken up in ethyl acetate (35 mL) and the solution was extracted with NaHCO_3(sat.)_ (3 × 50 mL), NaOH 1 M (3 × 50 mL) and, finally, HCl 1 M (1 × 30 mL). The organic layer was dried over Na_2_SO_4_ and concentrated to ca. ¼ of the starting volume by rotary evaporation; addition of petroleum ether (100 mL) afforded the title compound as a white powder that was recovered by filtration and further purified by recrystallization from DCM/petroleum ether (3:7) at −20 °C. Yield 75%. The monomer was stored under a nitrogen atmosphere at 4 °C in the dark to avoid oxidation of the aldehyde group.

ATR-IR cm^−1^: 2980 (w), 2937 (w), 2839 (w), 1810 (w), 1734 (s), 1689 (s), 1636 (w), 1597 (m), 1540 (w), 1502 (s), 1458 (m), 1421 (m), 1390 (m), 1376 (m), 1317 (m), 1285 (s), 1265 (s), 1241 (s), 1202 (s), 1147 (s), 1109 (s), 1029 (s), 1005 (s), 945 (s), 901 (w), 871.5 (m), 808.4 (m), 779.1 (s), 732.7 (s), 646.9 (m).

^1^H NMR (CDCl_3_) δ: 9.89 (s, 1H, -C*H*O); 7.43 (m, 2H, Ar-*H*); 7.20 (m, 1H, Ar-*H*); 6.31 (s, 1H, =C*H*); 5.73 (s, 1H, =C*H*); 3.83 (s, 3H, -OC*H*_3_); 2.00 (s, 3H, -C*H*_3_).

The spectroscopic data matched those reported in the literature [24].

### 2.4. General Procedure for the ARGET-ATRP of VMA

In a typical procedure, a degassed and N_2_ backfilled Schlenk flask equipped with a magnetic stir bar was charged with anisole (1 mL, 1/1 *v*/*wt* with respect to monomer), CuX_2_ (0.5 M in DMF, 1 equiv.), ligand (0.5 M in anisole, 1-2 equiv.), **VMA** (1.0 g, 4.5 mmol, 1000 equiv.), EBiB (neat, 5 equiv.) and Sn(EH)_2_ (neat, 10 equiv.). The flask was placed in an oil bath thermostated at 60 °C and stirred at 500 rpm for the desired time. The reaction was quenched upon venting the vessel, the crude mixture was diluted with dichloromethane (2 mL) and the product was precipitated in 2-propanol (50 mL), recovered by filtration and dried at 30 °C in vacuum for 16 h. Samples for biological testing were triturated 3 times with 2-propanol to remove excess monomer/Cu salts, recovered by filtration and dried in a vacuum at 30 °C for 16 h before use.

ATR-IR cm^−1^: 2939 (w), 2837 (w), 2733 (w), 1809 (w), 1754 (s), 1698 (s), 1595 (m), 1501 (s), 1464 (m), 1422 (m), 1389 (m), 1321 (w), 1268 (s), 1200 (m), 1082 (s), 1029 (s), 959.2 (w), 870.6 (m), 779.3 (m), 733.2 (s), 646.9 (w).

^1^H NMR (CDCl_3_) δ: 9.80 (bs, 1H, -C*H*O); 7.32 (m, 2H, Ar-*H*); 7.24 (m, 1H, Ar-*H*); 3.67 (bs, 3H, -OC*H*_3_).

### 2.5. Evaluation of the Antimicrobial Activity of Poly(Vanillin Methacrylate) (PVMA)

Polymer films (13 mm in diameter, 25 mg of material) for antimicrobial tests were obtained by solvent casting as follows: 130 µL of a stock solution of **PVMA** in dichloromethane (190 mg/mL) were placed in a PP mould and the solvent was allowed to slowly evaporate at room temperature. The films were further dried in a vacuum oven at 30 °C and 5 mbar for 24 h.

Gram-negative (*Escherichia coli* C1a) and Gram-positive (*Bacillus subtilis* ATCC 6051) bacterial strains were used to evaluate the antimicrobial activity in vitro of **PVMA**.

Bacteria were inoculated into Luria–Bertani (LB) broth, composed of 1% tryptone, 0.5% yeast extract, and 0.5% NaCl, and grown overnight (approximately 16 h) at 37 °C under constant shaking (180 rpm) in the dark condition. Bacterial growth was monitored spectrophotometrically by measuring the optical density at 600 nm (OD_600_) using a JASCO V-560 spectrophotometer.

The antimicrobial activity of **PVMA** samples in different forms was assessed using a broth inhibition bioassay [38]. Before testing, *E. coli* and *B. subtilis* cultures were diluted in LB broth to a final concentration of 10^6^ CFU/mL.

One millilitre of bacterial suspension was added to sterile VMA samples consisting of either 25 mg of **PVMA** powder or polymer films (13 mm in diameter) not hydrated, or pre-hydrated for 7 days, at 25 °C, in ultra-pure sterile water (Milli-Q). Samples were incubated for 3 h at 37 °C under shaking (120 rpm). Following incubation, 100 μL of each sample was transferred into wells of a 96-well MicroWell™ plate and subjected to a series of dilutions using phosphate buffer (61.4 mM K_2_HPO_4_, 38.4 mM KH_2_PO_4_). Each dilution (10 μL) was plated onto LB agar plates and incubated overnight at 37 °C. After incubation, bacterial colonies were counted to determine the number of viable cells (CFU/mL).

Control samples consisted of bacterial suspensions incubated under the same conditions in the absence of the polymer. Antibacterial activity was expressed as CFU/mL and as the percentage of bacterial survival relative to the control. All experiments were performed in triplicate.

## 3. Results and Discussion

### 3.1. VMA Synthesis and Characterization

The monomer, vanillin methacrylate (**VMA**), was synthetized via esterification of vanillin (**VA**) with a slight excess of methacrylic anhydride (**MAh**) in the presence of a catalytic amount of DMAP under solvent-free condition; since the multiple base treatment required by the previosly reported protocols [24] for the removal of the unreacted **MAh** proved in many attempts inefficient, alternative purification procedures were tested. Recrystallization of the crude, waxy mixture from DCM/petroleum ether (3:7 *v*/*v*) afforded **MAh**-free **VMA**, as confirmed by NMR spectroscopy analysis (see the Supporting Information, Figure S1). The yield of the reaction was 78%. To prevent its possible oxidation [39], the monomer was stored under a nitrogen atmosphere at 4 °C in the dark.

### 3.2. ARGET-ATRP of VMA—Screening of the Reaction Conditions

**Scheme 1:**
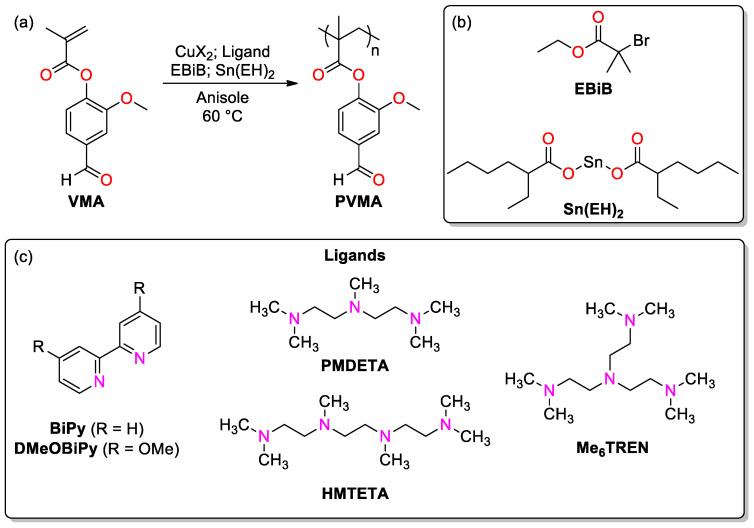
ARGET-ATRP of **VMA** (**a**); structures of the initiator and of the reducing agent (**b**); structures of the ligands (**c**).

The polymerization of **VMA** by ARGET-ATRP was next investigated. Preliminary tests involving CuBr_2_ and CuCl_2_ as the metal source were carried out in anisole with a monomer:Cu ratio of 2000:1 (corresponding to 500 ppm of the metal) in the presence of 50 equivalents (with respect to copper) of the reducing agent (**Sn(EH)_2_**) and with **EBiB** as the initiator. In terms of ligands, both aromatic (**BiPy** and **DMeOBiPy**) and aliphatic (**PMDETA**, **HMTETA** and **Me6TREN**) amines were considered (Scheme 1). In order to avoid the involvement of the aldehyde group in side processes (i.e., polyacetal formation [40]), the temperature was set at the compromise value of 60 °C. In fact, reactions performed in solvent-free conditions and/or at higher temperatures afforded cross-linked materials insoluble in common organic solvents (see the Supporting Information, Table S1). Catalysts involving bipyridine-based ligands proved less performing than their aliphatic amine-bearing counterparts; moreover, unlike the reactivity trends commonly observed for methacrylic monomers, CuCl_2_-based complexes allowed for higher conversions than their CuBr_2_ congeners [41]. Although counterintuitive, this evidence reflects the lower activity of the chlorinated systems in linear chain propagation; indeed, in this case, the reduced activity likely promotes side reactions involving the aldehyde, thereby leading to higher overall conversions, albeit to the formation of cross-linked, insoluble materials, rather than linear polymers. In fact, aside from the reactions performed with **DMeOBiPy** and CuBr_2_/**BiPy** systems, all tests were interrupted due to the formation of insoluble products, hampering the stirring of the mixture.

Based on this evidence, it was proposed that, in spite of the high conversions reached in most cases, the reaction conditions were not suitable for granting a controlled process, hence the preservation of the aldehyde function. This was tentatively accounted to the high concentrations of the initiator (**EBiB**) producing a large number of radicals [28,29] and/or to the presence of excess of **Sn(EH)_2_** able to activate carbonyl compounds (as in the ROP of lactones [42]), making the aldehyde function more prone to participate in side-processes. On the other hand, tests performed in the presence of alternative reducing agents, namely ascorbic acid and glucose, proved unsuccessful (see the Supporting Information, Tables S2 and S3). Milder reaction conditions (still involving **Sn(EH)_2_**) were thus investigated (Table 1). While solvent and temperature were not modified, the relative amounts of initiator and reducing agent were lowered (5 vs. 10 equiv. and 10 vs. 50 equiv., respectively) and, simultaneously, the catalyst loading was increased (1000 vs. 500 ppm). Due to the ineffectiveness of **DMeOBiPy** under harsher reaction conditions, its employment was not further considered.

**Scheme 2:**
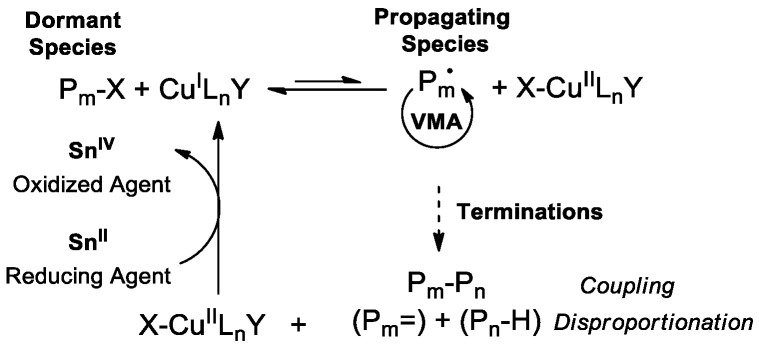
Representation of the ARGET-ATRP of **VMA**.

The catalyst activity followed the trend **HMTETA** > **PMDETA** >> **BiPy**, which is in agreement with the *K*_ATRP_ values determined for such ligands in classical ATRP [43,44]. Indeed, when CuBr_2_ was employed, **HMTETA** led to the highest monomer conversion, reaching 28% within 6 h, while **PMDETA** afforded a slightly lower conversion of 20% under the same conditions. While the **BiPy**-based catalyst proved inactive regardless of the copper salt, for both **PMDETA** and **HMTETA,** higher conversions were attained in the presence of CuBr_2_ instead of CuCl_2_. This behaviour can be explained by the different strengths of the halogen–copper and halogen–carbon bonds influencing the activation-deactivation equilibrium of ATRP (Scheme 2). It has been shown that in a mixed environment (CuCl and Br-containing initiator, namely **EBiB**), the dormant P_m_-X species would preferentially feature chloride over bromide [45,46]. As a consequence, the concentration of the propagating radical would be lower due to the stronger C–Cl bonds (left-shift in the equilibrium), leading to slower polymerization rates and lower final conversions.

THF-soluble polymers with narrow polydispersities were attained upon employing **PMDETA** as the ligand (Table 1, entries 1–4 and Supporting Information, Figures S2–S5). In all cases, experimental *M*_n_ were found to be higher than the theoretical values, suggesting the incomplete activation of the initiator. Nevertheless, this has to be considered only as indicative, since the values have been determined against PS standards and could not be corrected with an appropriate Mark-Houwink coefficient. Conversely, no signals accountable to polymer species were observed on the SEC traces of the samples isolated from the tests involving **HMTETA** (Table 1, entries 5–8). This could be ascribed to the formation of oligomers having *M*_n_ too low to be detected. Nevertheless, the formation of THF-insoluble cross-linked polymers due to a high radical concentration generated by the highly active **HMTETA**-based catalysts could not be ruled out. In light of these results, the CuBr_2_-**PMDETA** system emerged as the right compromise between monomer conversion and process control.

### 3.3. ARGET-ATRP of VMA—Kinetics Investigations

**Figure 1:**
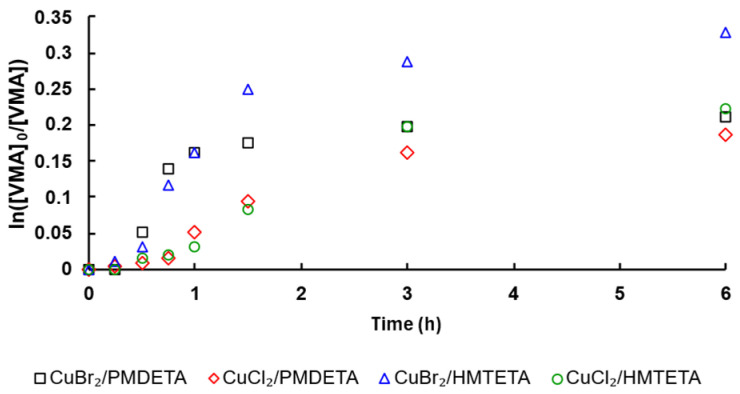
Kinetics profiles for the Cu-catalyzed ARGET-ATRP of **VMA**.

Kinetic investigations were conducted upon employing **PMDETA** and **HMTETA** as the ligands (Figure 1). Aside from being overall less performing than their bromide analogues, CuCl_2_-based catalysts exhibited a short induction period. CuBr_2_/**HMTETA** allowed for the highest rate and linearity during the early stages of the process, although the variation in *M*_n_ and *Ɖ* with the monomer conversion could not be assessed (vide supra).

**Figure 2:**
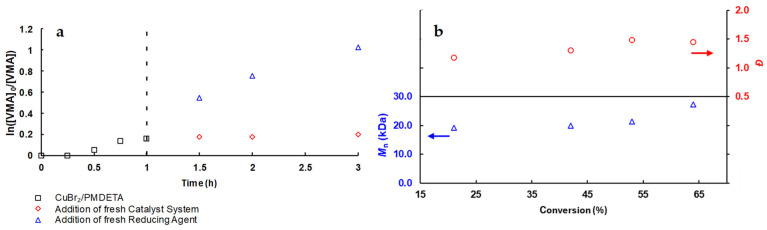
Influence of freshly added catalyst system or reducing agent on the monomer conversion *plateau* (**a**) and dependency of *M*_n_ and *Ɖ* on the monomer conversion after reactivation of the systems with the reducing agent (**b**).

Regardless of the system employed, a conversion *plateau*, accountable to catalyst deactivation and/or depletion of the reducing agent, was observed after 3 h. To shed light on the origin of such a phenomenon, a reactivation experiment was carried out by adopting the CuBr_2_/**PMDETA** complex as the model catalyst system. Two parallel polymerizations were monitored, and after 1 h, either the catalytic complex or the reducing agent (**Sn(EH)_2_**) was added in the same amounts as initially employed (Figure 2a).

Upon addition of *fresh* copper catalyst, no effect on the **VMA** conversion was observed, while the introduction of further 10 equivalents of **Sn(EH)_2_** led to a significant increase in the monomer conversion, reaching 63% within 3 h. These experiments suggested that the conversion *plateau* could be due to the depletion of the reducing agent involved in the reactivation of accumulated Cu(II) originated from termination processes (i.e., coupling, disproportionation, vide supra) that could no longer be reduced due to the progressive consumption of **Sn(EH)_2_**. The test involving the freshly added Cu(II)-based species, indeed, indicated the absence of residual Sn(II) still capable of regenerating the catalyst system. A similar behaviour was observed in the ARGET-ATRP of eugenyl methacrylate [8]. In addition, after reactivation, a progressive linear increase in *M*_n_ with the monomer conversion was observed (Figure 2b), indicating, in spite of the *plateau*, the presence of polymer chains still bearing active termini. It has to be noted that, although a slight broadening of the molecular weight distribution was observed, the largest value measured after 24 h from the reactivation was still lower than 1.5, suggesting good polymerization control even after reactivation.

### 3.4. ARGET-ATRP of VMA—Polymer Characterization

The ^1^H NMR spectrum (see the Supporting Information, Figure S6) of the polymer isolated with the CuBr_2_/**PMDETA** system (Table 1, entry 2) showed all resonances accountable to **PVMA,** and the ratios between their relative integrations were in good agreement with expected values, indicating the preservation of the aldehyde group throughout the polymerization process. The comparison of the areas of the signals at 1.59, 1.57 and 1.49 ppm, corresponding to the *mm*, *mr* and *rr* triads, respectively (with *m* = meso and *r* = racemo), suggested that the polymers, although essentially atactic, exhibited a slight syndiotactic bias, as expected in radical, non-stereospecific polymerizations [47].

The DSC measurement on the same sample indicated a glass transition temperature (*T*_g_) of ca. 104 °C (see the Supporting Information, Figure S7), in line with previous reports [25]. Given that the *T*_g_ of poly(styrene) (PS) is generally found in the range 97–107 °C [48], PVMA could be considered as a potential bio-based replacement for such ubiquitous, fossil-based thermoplastic. The TGA trace of the material (see the Supporting Information, Figure S8) indicated an onset temperature of ca. 230 °C, somewhat lower than that exhibited by PS and poly(methyl methacrylate) (PMMA) (350 and 330 °C, respectively [49,50]); on the other hand, the decomposition pattern, as well as the lower *T_onset_* (compared to that of PS and PMMA) were in agreement with previous reports on this type of bio-derived phenol methacrylic polymers [51].

**Figure 3:**
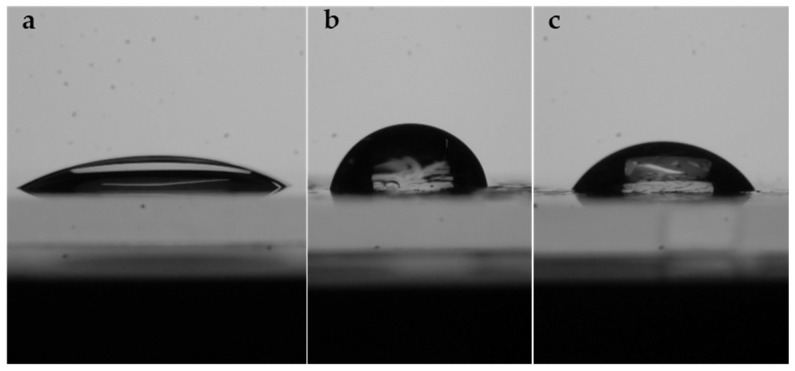
Comparison between the wettability of neat glass surface ((**a**), CA = 29°), **PVMA** film ((**b**), CA = 83°) and **PVMA** film subjected to hydration treatment ((**c**), CA = 58°).

The wettability of **PVMA** was quantified by contact angle (CA) measurements (Figure 3). With respect to a neat glass surface, the polymer exhibited significantly lower hydrophilicity (CA values of 29° and 83°, respectively, Figure 3a,b). Furthermore, aiming at enhancing the polarity of the surface by increasing its polar group density (i.e., carbonyls), polymer films were subjected to pre-hydration in ultra-pure water for 7 days at 25 °C and subsequently dried under vacuum at 30 °C for 16 h. Interestingly, the CA (58°, Figure 3c) was lower than that measured for the non-treated film, indicating a higher hydrophilic character. It was proposed that exposure to water led to a deformation of the structure of the **PVMA** layer, causing an increase in the density of surface aldehyde groups and, consequently, a higher surface polarity [52,53].

Notably, the film proved stable under the hydration conditions; indeed, no signals related to oxidation products (i.e., carboxylic acid) were observed in the IR- and ^1^H NMR spectra of the treated sample (See the Supporting Information, Figures S9 and S10, respectively).

### 3.5. Antimicrobial Potential of PVMA

**Figure 4:**
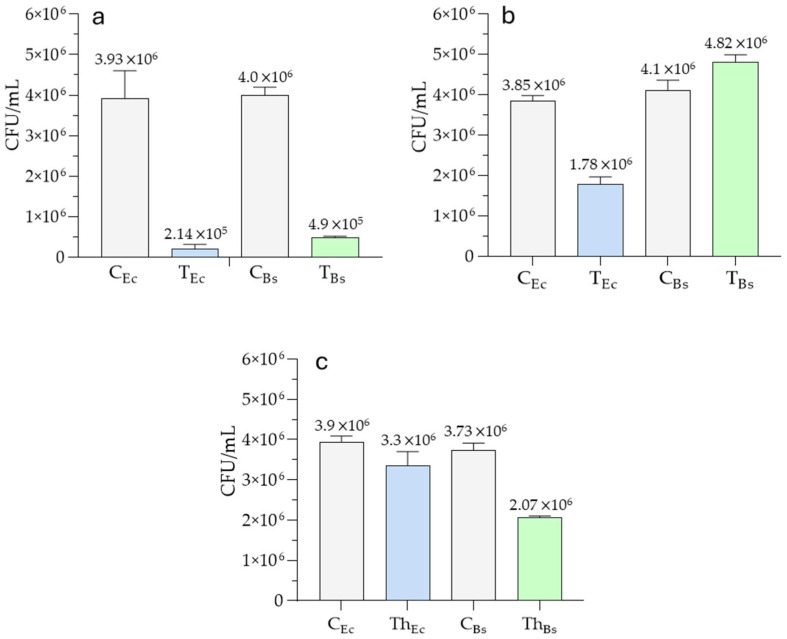
Antimicrobial activity of **PVMA**. Activity of **PVMA** powder (**a**); activity of **PVMA** polymeric film (**b**); activity of pre-hydrated **PVMA** polymeric film (**c**). C_Ec_: *E. coli* control; T_Ec_: *E. coli* PVMA- treated; C_Bs_: *B. subtilis* control; T_Bs_: *B. subtilis* PVMA-treated; Th_Ec_: *E. coli* pre-hydrated PVMA-treated; Th_Bs_: *B. subtilis* pre-hydrated **PVMA**-treated.

Antimicrobial activity assays of PVMA were conducted in vitro against the Gram-negative bacterium *E. coli* and the Gram-positive bacterium *B. subtilis*. The absence of metal traces from the samples employed in the antimicrobial tests was confirmed by SEM-EDX analysis (see the Supporting Information, Figure S11). In the first experiment, PVMA was tested in powder form (Figure 4, left). The results demonstrated high antimicrobial efficacy against both *E. coli* (T_Ec_) and *B. subtilis* (T_Bs_), with survival rates of 5.4% and 12.2%, respectively, relative to the corresponding control samples (C_Ec_ and C_Bs_). In the second series of experiments, PVMA was evaluated in the form of a film (Figure 4, right). Under these conditions, antimicrobial activity was observed only against *E. coli* (T_Ec_), with a survival rate of 46.2%. In contrast, no antimicrobial effect was detected against *B. subtilis* (TBs). Finally, the **PVMA** films previously subjected to the hydration treatment (vide supra) were tested; unlike the non-treated films, the material proved higher activity against *B. Subtilis* than *E. Coli* (survival rate of 55.5 and 84.6%, respectively).

The greater efficiency of the powder was tentatively accounted to the number of aldehyde groups available to interact with bacteria walls. With respect to the films, antimicrobial action occurs in two steps: bacterial adhesion to the surface (i) and interaction with the exposed biocidal functions (ii) [54]. Possibly, the polarity of the surface plays a role in the first step, differentiating the activity of the material based on the nature of the bacterial wall.

It is well known that vanillin acts as a membrane-perforating compound, inhibiting the growth of various Gram-negative and Gram-positive bacteria by destroying their cell membranes [55,56,57]. However, to date, relatively few studies have investigated the precise mechanism by which vanillin damages the molecular architecture of bacterial membranes; therefore, specific targets and mechanisms of action remain unclear. So, further studies are currently underway to thoroughly investigate the mechanisms underlying the interaction between the polymer and bacterial walls.

## 4. Conclusions

In this communication, we reported the unprecedented systematic investigation of the polymerization of the bio-derived monomer vanillin methacrylate (**VMA**) by ARGET-ATRP. Optimization of the reaction conditions aimed at preserving the aldehydic function was carried out, and further investigations proved that, amongst those explored, CuBr_2_/**HMTETA** was the most active catalyst system while better control was attained in the presence of its **PMDETA**-bearing analogue. Kinetics studies revealed, for all catalysts, linear profiles within the first 1.5 h of polymerization before reaching a conversion plateau caused by the depletion of the reducing agent involved in the reactivation of the *spent* metal catalyst, as suggested by reactivation experiments. The isolated materials exhibited *T*_g_ values comparable to that of the ubiquitous, fossil-based, poly(styrene) and, thanks to the preservation of the aldehydic groups during the polymerization process, polymer powder displayed antimicrobial activity against both Gram-positive and Gram-negative bacteria while films exhibited different potential depending on the polarity of the surface allegedly derived from the number of accessible aldehyde groups. In conclusion, ARGET-ATRP proved effective under milder conditions compared to previously reported controlled radical polymerization methods for PVMA, such as RAFT and RITP, without requiring extensive purification of commercially available reactants and/or strictly anhydrous conditions. Moreover, the control over the radical species concentration allowed for the preservation of the aldehyde function, making this technique particularly useful for the preparation of materials with antimicrobial potential. In terms of polymer features, the present approach produced **PVMA** with narrower dispersities than those reported for other controlled polymerization techniques, albeit with lower *M*_n_. To overcome such limitations, further optimisations, aiming at increasing the control over the termination events, thus opening the possible preparation of well-defined architectures (i.e., block copolymers), are currently ongoing in our laboratories.

## Figures and Tables

**Table 1:** ARGET-ATRP of **VMA** with 1000 ppm of Cu.

Entry	VMA (equiv.)	CuX_2_ (equiv.)	Ligand (equiv.)	Time (h)	Conv. (%) *^a^*	*M*_n_ (kDa) *^b^*	*Ɖ* *^b^*
1	1000	CuBr_2_ (1)	**PMDETA** (1)	1	15	15.9	1.17
2				6	20	17.8	1.18
3		CuCl_2_ (1)		1	4	7.7	1.12
4				6	17	12.6	1.08
5		CuBr_2_ (1)	**HMTETA** (1)	1	15	*n.d.*	
6				6	28		
7		CuCl_2_ (1)		1	3		
8				6	20		
9		CuBr_2_ (1)	**BiPy** (2)	24	nil	-	-
10		CuCl_2_ (1)				-	-

**Reaction conditions:** Anisole 1 mL, **VMA** 1.0 g (1000 equiv.), **CuX_2_** (0.4 M in DMF, 1 equiv., 1000 ppm), ligand (0.4 M in anisole, 1-2 equiv.), **EBiB** (neat, 5 equiv.), **Sn(EH)_2_** (neat, 10 equiv.) *^a^* Determined by ^1^H NMR spectroscopy on the crude reaction mixture. *^b^* Determined by GPC at 30 °C in THF against PS standards. *n.d. = not detectable.*

## Data Availability

The original contributions presented in this study are included in the article/Supplementary Material. Further inquiries can be directed to the corresponding author.

## Supplementary Materials

The following supporting information can be downloaded at: https://www.mdpi.com/article/10.3390/polym18091023/s1, Scheme S1. Synthesis of vanillin methacrylate (VMA). Figure S1. Comparison between the ^1^H NMR spectra (CDCl_3_, 400 MHz, 298 K) of VMA methacrylate before (left) and after (right) recrystallization from DCM/petroleum ether. Figure S2. SEC trace of PVMA synthesized in Table 1, entry 1. Figure S3. SEC trace of PVMA synthesized in Table 1, entry 2. Figure S4. SEC trace of PVMA synthesized in Table 1, entry 3. Figure S5. SEC trace of PVMA synthesized in Table 1, entry 4. Figure S6. ^1^H NMR spectrum (CDCl_3_, 400 MHz, 298 K) of PVMA (Table 1 entry 2). *m* = meso and *r* = racemo triads. Figure S7. DSC trace (2nd heating ramp) of PVMA (Table 1, entry 2) indicating the glass transition temperature (*T*_g_). Figure S8. TGA trace of PVMA (Table 1, entry 2) indicating onset temperature of 227 °C and a residual 7% weight at 600 °C. Figure S9. Comparison between the FT-IR spectra of PVMA (Table 1, entry 2) before and after hydration treatment. Figure S10. Comparison between the ^1^H NMR spectra of PVMA (Table 1, entry 2) before and after pre-hydration treatment. Figure S11. SEM-EDX analysis of the PVMA employed in antimicrobial tests indicating negligible presence of metal traces. Table S1. Preliminary catalyst systems screening for the ARGET-ATRP of VMA. Table S2. ARGET-ATRP of VMA at higher temperature and/or in bulk. Table S3. ARGET-ATRP of VMA in the presence of Ascorbic Acid as the reducing agent. Table S4. ARGET-ATRP of VMA in the presence of glucose as the reducing agent.
