# Supplementary material for: Bio-Based Polymers with Potential Antimicrobial Activity from Vanillin Methacrylate via ARGET-ATRP
Source: Polymers (Basel). 2026 Apr 23;18(9):1023. doi: 10.3390/polym18091023 (PMC13164883; doi:10.3390/polym18091023)
Supplement: Supplementary file 1 [file polymers-18-01023-s001.zip › polymers-4237063-supplementary.pdf]

Supporting Information

for

Bio-based polymers with potential antimicrobial activity from Vanillin Methacrylate *via* ARGET-ATRP

Eddy Marelli[1], Maristella Mastore[2], Maurizio F. Brivio[2], Francesco Della Monica[1], Lorella Izzo[1], Orlando Santoro[1,*]

[1] Laboratory of Polymer Chemistry and Sustainable Catalysis, Department of Biotechnology and Life Sciences (DBSV), University of Insubria, 21100 Varese, Italy

[2] Laboratory of Applied Entomology and Parasitology, Department of Theoretical and Applied Sciences (DiSTA), University of Insubria, 21100 Varese, Italy

*orlando.santoro@uninsubria.it

Contents

Synthesis and characterization of Vanillin Methacrylate (VMA) S3

Scheme S1. *Synthesis of vanillin methacrylate (**VMA**)* S3

Figure S1. *Comparison between the ^{1}H NMR spectra ($CDCl_3$, 400 MHz, 298 K) of **VMA** methacrylate before (left) and after (right) recrystallization from DCM/petroleum ether.* S3

ARGET-ATRP of VMA S4

Screening of reaction conditions for the ARGET-ATRP of VMA S4

Table S1. *Preliminary catalyst systems screening for the ARGET-ATRP of **VMA**.* S4

Table S2. *ARGET-ATRP of **VMA** at higher temperature and/or in bulk* S4

Screening of reducing agents for the ARGET-ATRP of VMA S5

Table S3. *ARGET-ATRP of **VMA** in the presence of Ascorbic Acid as the reducing agent.* S5

Table S4. *ARGET-ATRP of **VMA** in the presence of glucose as the reducing agent.* S5

Characterization of PVMA S6

Figure S2. *SEC trace of **PVMA** synthesized in Table 1, entry 1.* S6

Figure S3. *SEC trace of **PVMA** synthesized in Table 1, entry 2.* S6

Figure S4. *SEC trace of **PVMA** synthesized in Table 1, entry 3.* S7

Figure S6. *^{1}H NMR spectrum ($CDCl_3$, 400 MHz, 298 K) of **PVMA** (table 1 entry 2).* S8

Figure S7. *DSC trace (2nd heating ramp) of **PVMA** (table 1, entry 2) indicating the glass transition temperature (T_g).* S8

Figure S8. *TGA trace of **PVMA** (table 1, entry 2) indicating onset temperature of 227 °C and a residual 7% weight at 600 °C.* S9

Pre-hydration of PVMA films S10

Figure S9. *Comparison between the FT-IR spectra of **PVMA** (table 1, entry 2) before and after pre-hydration treatment.* S10

Figure S10. *Comparison between the ^{1}H NMR spectra of **PVMA** (table 1, entry 2) before and after pre-hydration treatment.* S10

PMVA characterization for Antimicrobial Tests S11

Figure S11. *SEM-EDX analysis of the PVMA employed in antimicrobial tests indicating negligible presence of metal traces.* S11

Synthesis and characterization of Vanillin Methacrylate (VMA)

Vanillin methacrylate (**VMA**) was synthetized via esterification of vanillin (**VA**) with a slight excess of methacrylic anhydride (**MAh**) in the presence of a catalytic amount of DMAP under solvent-free condition (Scheme S1) [ref]. The crude, waxy, mixture was purified by recrystallization form DCM/petroleum ether (3:7 v/v) affording **MAh**-free **VMA**, as indicated by NMR spectroscopy analysis (Figure *S*1).

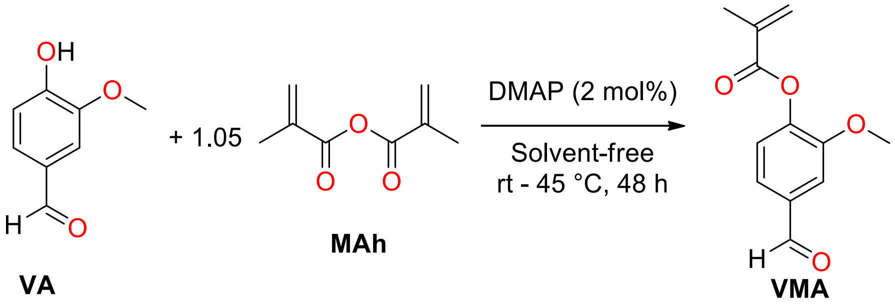

Scheme S1. *Synthesis of vanillin methacrylate (**VMA**)*

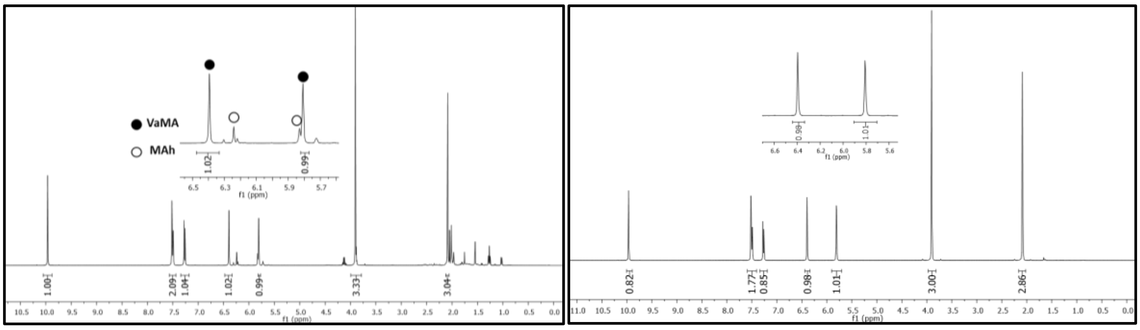

Figure S1. *Comparison between the* ^{1}H *NMR spectra* ($CDCl_3$, *400 MHz, 298 K) of* ***VMA*** *methacrylate before (left) and after (right) recrystallization from DCM/petroleum ether.*

ARGET-ATRP of VMA

Screening of reaction conditions for the ARGET-ATRP of VMA

Table S1. *Preliminary catalyst systems screening for the ARGET-ATRP of **VMA**.*

Entry	VMA (equiv.)	T (°C)	CuX_2 (equiv.)	Ligand (equiv.)	Time (h)	Yield (%)[a]
1	2000	60	$CuBr_2$ (1)	**PMDETA** (1)	2.5	75
2					1.0	47
3			$CuCl_2$ (1)		1.5	97
4					1.0	60
5			$CuBr_2$ (1)	**BiPy** (2)	24	30
6			$CuCl_2$ (1)		4	63
7			$CuBr_2$ (1)	**DMeOBiPy** (2)	24	15
8			$CuCl_2$ (1)		24	5
9			$CuBr_2$ (1)	**HMTETA** (1)	1.5	86
10			$CuCl_2$ (1)		0.75	72
11			$CuBr_2$ (1)	**Me6TREN** (1)	1.5	94
12			$CuCl_2$ (1)		0.75	84

Reaction conditions: Anisole 1 mL, **VMA** 1.0 g (2000 equiv.), CuX_2 (0.4 M in DMF, 1 equiv.), ligand (0.4 M in anisole, 1-2 equiv.), **EBiB** (neat, 10 equiv.), **Sn(EH)$_2$** (neat, 50 equiv.) [a] Determined gravimetrically.

Table S2. *ARGET-ATRP of **VMA** at higher temperature and/or in bulk*

Entry	CuX_2	Ligand	Time (h)	T (°C)	Yield (%)[a]
1	$CuBr_2$	BiPy	84	60	4.0
2			18	90	11
3			23		10
4[b]			1		20[c]
5[b]		PMDETA	7 min		77[c]
6			4		12
7			8		50
8			18		60
9			24		60
10			24		2.5
11	$CuCl_2$	PMDETA	24	90	1.3

Reaction conditions: Anisole 1 mL, **VMA** 1.0 g (800 equiv.), CuX_2 1 equiv., Ligand 2 equiv., EBiB 10 equiv., Sn(EH)$_2$ 50 equiv. [a] Determined by: [(polymer mass)/(monomer mass)]x100. [b] Reaction performed in solvent-free conditions. [c] Stirring stopped.

Screening of reducing agents for the ARGET-ATRP of VMA

Reducing agents alternative to $Sn(EH)_2$ were tested, namely Ascorbic Acid and Glucose.

Albeit good conversions were attained in the presence of ascorbic acid (Table S3), reproducibility issues, tentatively accounted to its poor solubility in the reactions medium, were observed. On the other hand, glucose prove completely inefficient (Table S4).

Table S3. *ARGET-ATRP of* ***VMA*** *in the presence of Ascorbic Acid as the reducing agent.*

Entry	CuX_2	Ligand	Time (h)	T (°C)	Yield (wt%)[a]	Ascorbic Acid (equiv.)
1[b]	$CuBr_2$	PMDETA	0.5	90	66	100
2[b]			1.5		45	100
3[b]			0.5		64	50
4			24		39	100
5			18		14	50
6	$CuCl_2$		18		4	100
7			24		/	100

Reaction conditions: Anisole 1 mL, **VMA** 1.0 g (800 equiv.), CuX_2 1 equiv., PMDETA 2 equiv., EBiB 10 equiv., Ascorbic acid 50 - 100 equiv. [a] Determined by: [(polymer mass)/(monomer mass)]x100. [b] Stirring stopped.

Table S4. *ARGET-ATRP of* ***VMA*** *in the presence of glucose as the reducing agent.*

Entry	CuX_2	Ligand	Time (h)	T (°C)	Yield (wt%)[a]	Glucose (equiv.)
1	$CuBr_2$	PMDETA	18	90	2	50
2					4	100

Reaction conditions: Anisole 1 mL, **VMA** 1.0 g (800 equiv.), CuX_2 1 equiv., PMDETA 2 equiv., EBiB 10 equiv., Glucose 50 - 100 equiv. [a] Determined by: [(polymer mass)/(monomer mass)]x100.

Characterization of PVMA

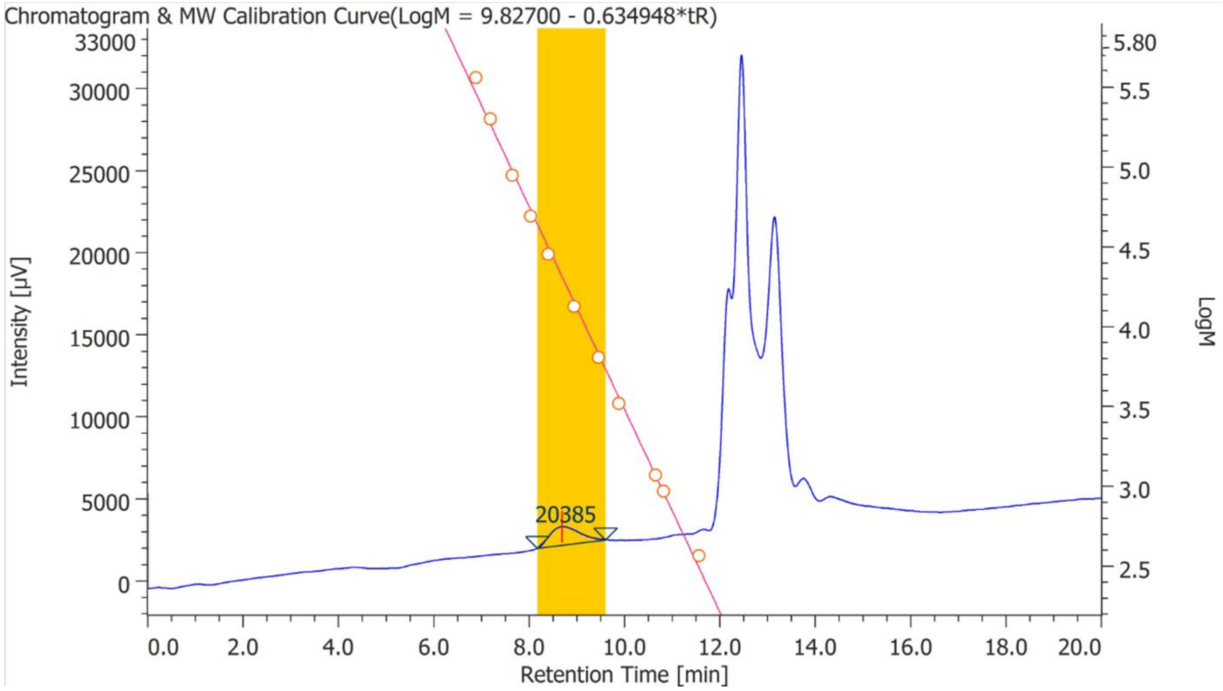

< MW Calculation Results(Section) >

#	Range	CH	Baseline Range	tR	Mp	Mn	Mw	Mz	Mv	Mw/Mn	Mz/Mw	Range Value	Percentage	Warning	Sample Name
1	8.1683 – 9.5983	1	8.1683 – 9.5983	8.6900	20385	16603	19524	22329	19524	1.1759	1.1437	49054	100.00		

Figure S2. *SEC trace of* ***PVMA*** *synthesized in Table 1, entry 1.*

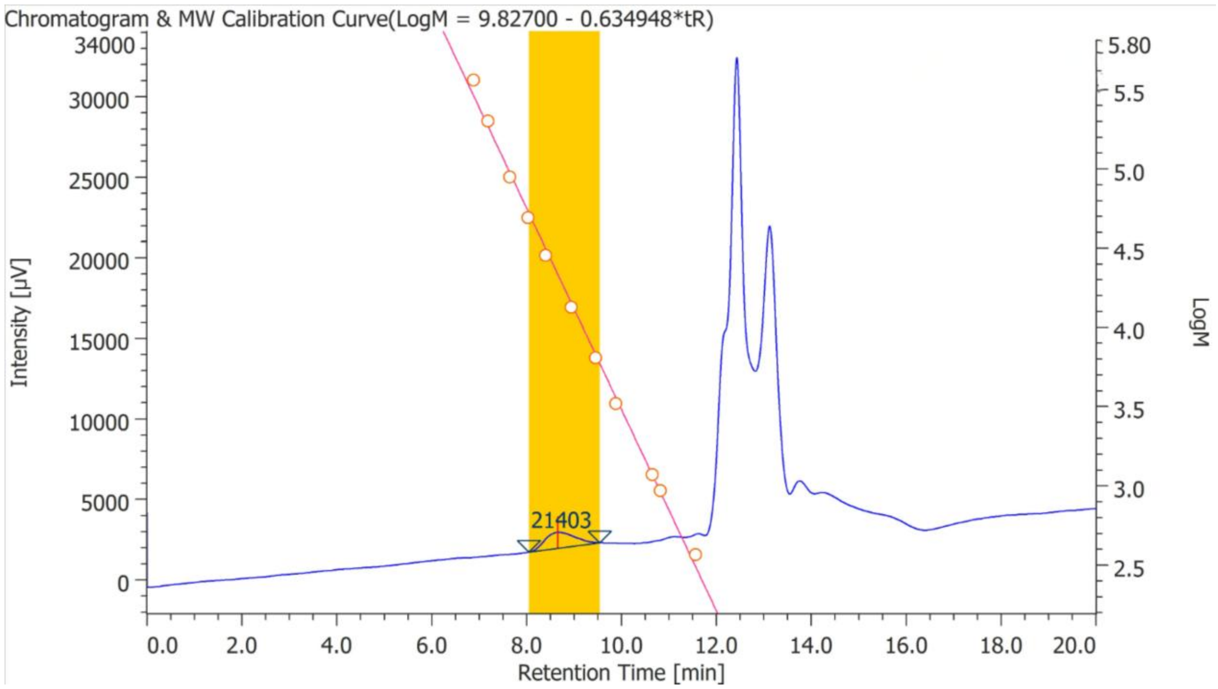

< MW Calculation Results(Section) >

#	Range	CH	Baseline Range	tR	Mp	Mn	Mw	Mz	Mv	Mw/Mn	Mz/Mw	Range Value	Percentage	Warning	Sample Name
1	8.0450 – 9.5367	1	8.0450 – 9.5367	8.6567	21403	17845	20967	24071	20967	1.1749	1.1481	43551	100.00		

Figure S3. *SEC trace of* ***PVMA*** *synthesized in Table 1, entry 2.*

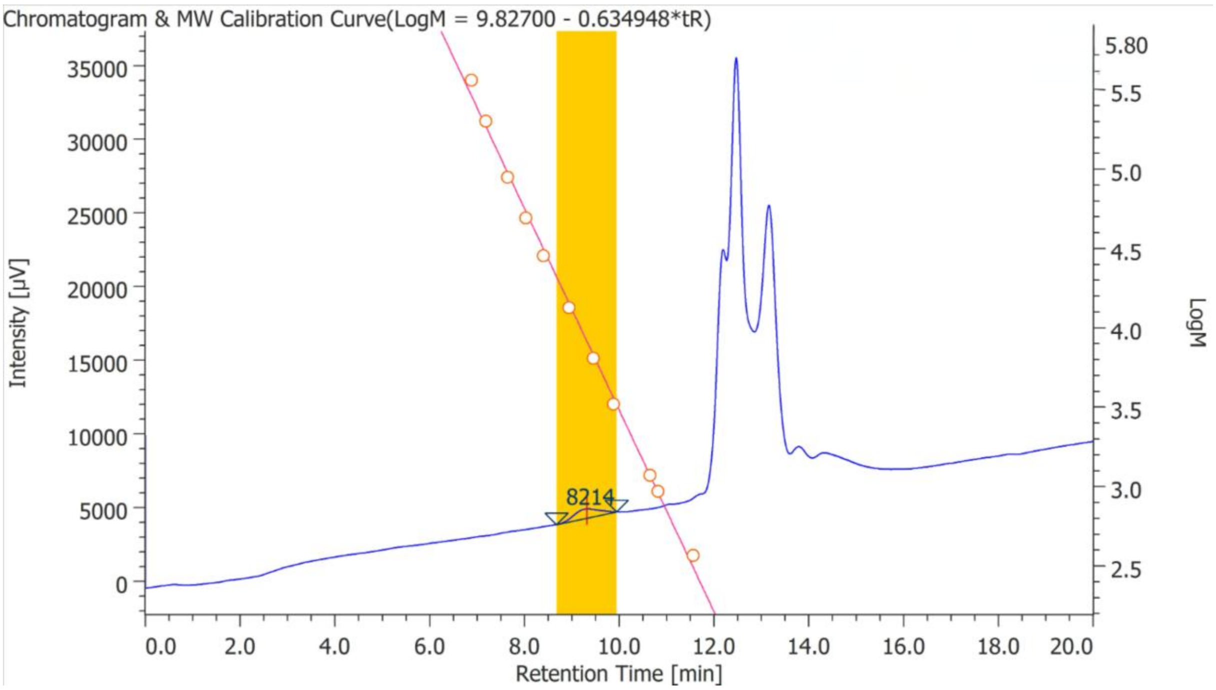

< MW Calculation Results(Section) >

#	Range	CH	Baseline Range	tR	Mp	Mn	Mw	Mz	Mv	Mw/Mn	Mz/Mw	Range Value	Percentage	Warning	Sample Name	Description
1	8.6733 – 9.9450	1	8.6733 – 9.9450	9.3117	8214	7658	8602	9566	8602	1.1232	1.1121	23924	100.00			

Figure S4. *SEC trace of* ***PVMA*** *synthesized in Table 1, entry 3.*

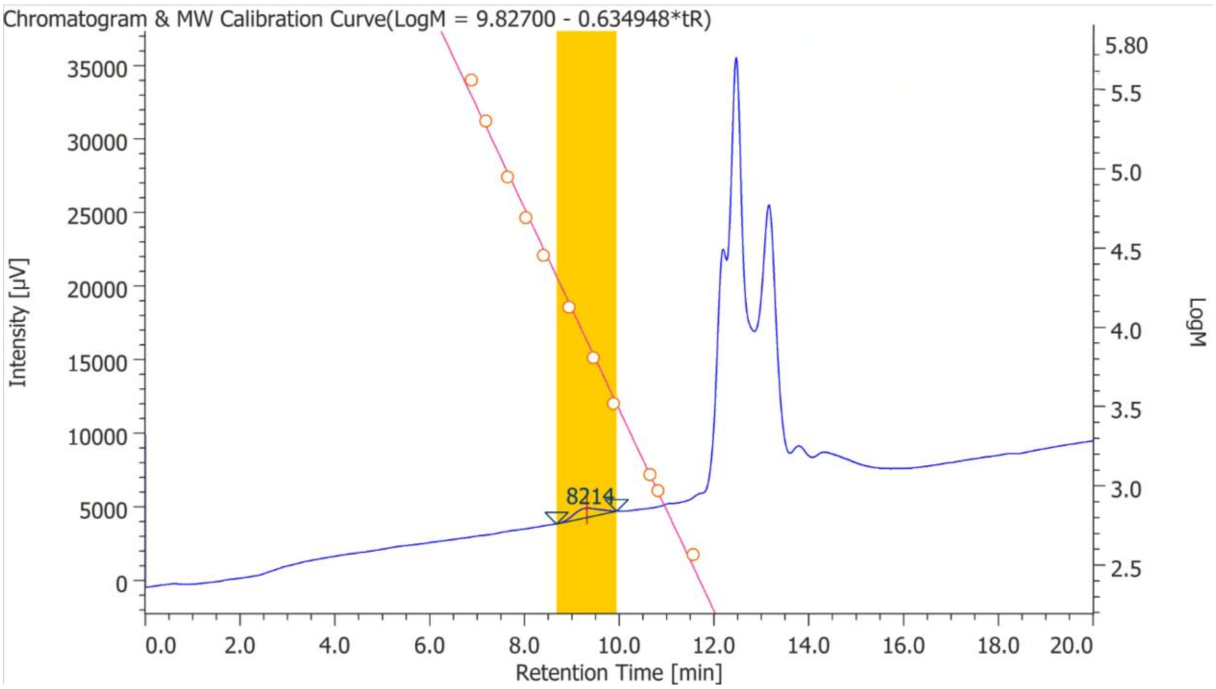

< MW Calculation Results(Section) >

#	Range	CH	Baseline Range	tR	Mp	Mn	Mw	Mz	Mv	Mw/Mn	Mz/Mw	Range Value	Percentage	Warning	Sample Name
1	8.5633 – 9.4750	1	8.5633 – 9.4750	9.0017	12924	12606	13484	14367	13484	1.0696	1.0655	19339	100.00		

Figure S5. *SEC trace of* ***PVMA*** *synthesized in Table 1, entry 4.*

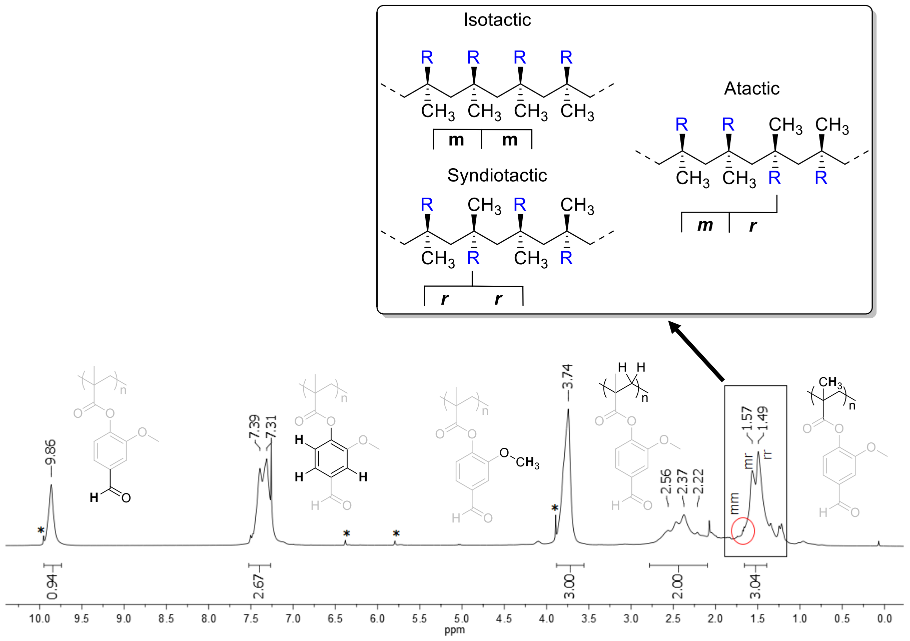

Figure S6. *^{1}H NMR spectrum (CDCl3, 400 MHz, 298 K) of* ***PVMA*** *(table 1 entry 2).*
*(m = meso and r = racemo triads). *Residual monomer (< 3mol%).*

Note: the ratio between the integrations of the signals at 9.86 and 3.74 ppm (-C*H*O and -OC*H*3, respectively) is in agreement with that determined for the same signals on the monomer spectrum, indicating preservation of the aldehyde function during the polymerization.

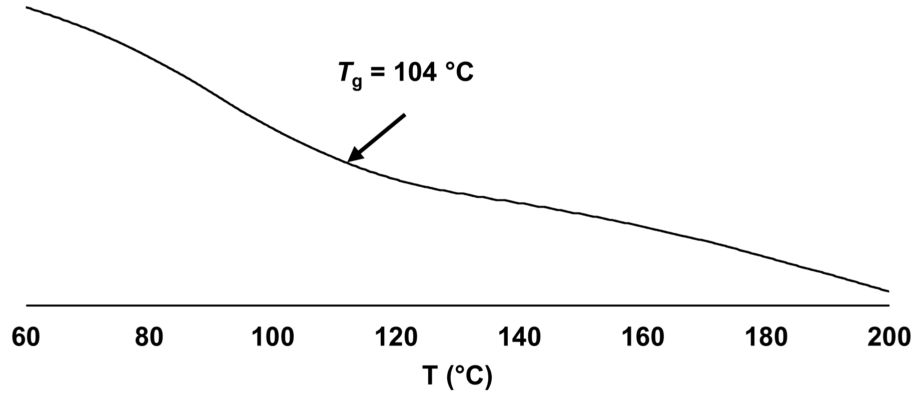

Figure S7. *DSC trace (2nd heating ramp) of* ***PVMA*** *(table 1, entry 2) indicating the glass transition temperature (T_g).*

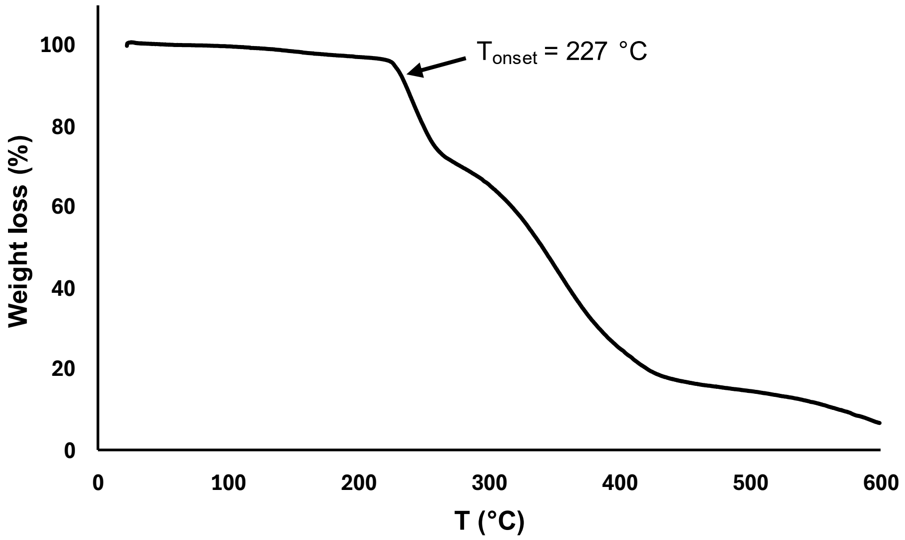

Figure S8. *TGA trace of* ***PVMA*** *(table 1, entry 2) indicating onset temperature of 227 °C and a residual 7% weight at 600 °C.*

Pre-hydration of PVMA films

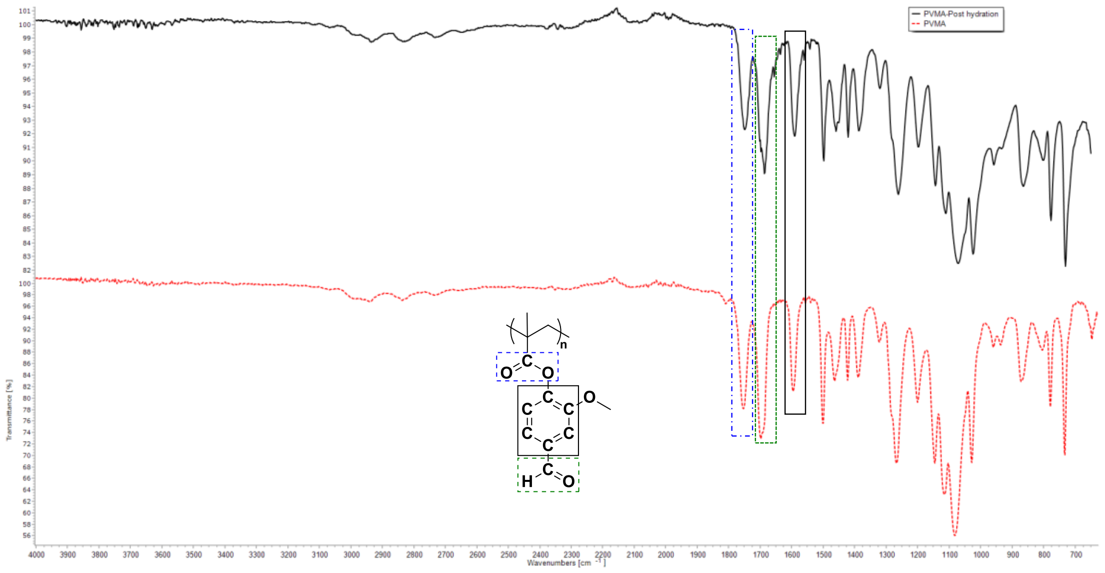

Figure S9. *Comparison between the FT-IR spectra of* ***PVMA*** *(table 1, entry 2) before and after pre-hydration treatment.*

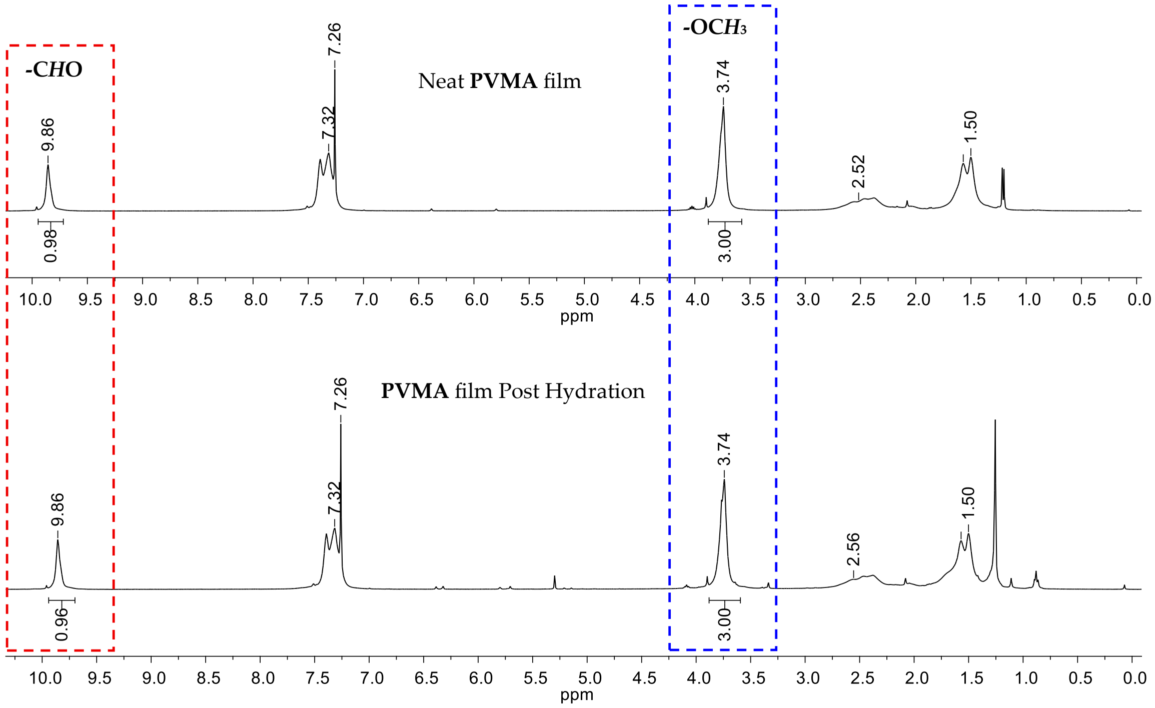

Figure S10. *Comparison between the* ^{1}H *NMR spectra of* ***PVMA*** *(table 1, entry 2) before and after pre-hydration treatment.*

Note: the ratios between the integrations of the signals at 9.86 and 3.74 ppm (-C*HO* and -OCH_3, respectively) are found in very narrow range, indicating preservation of the aldehyde function.

PMVA characterization for Antimicrobial Tests

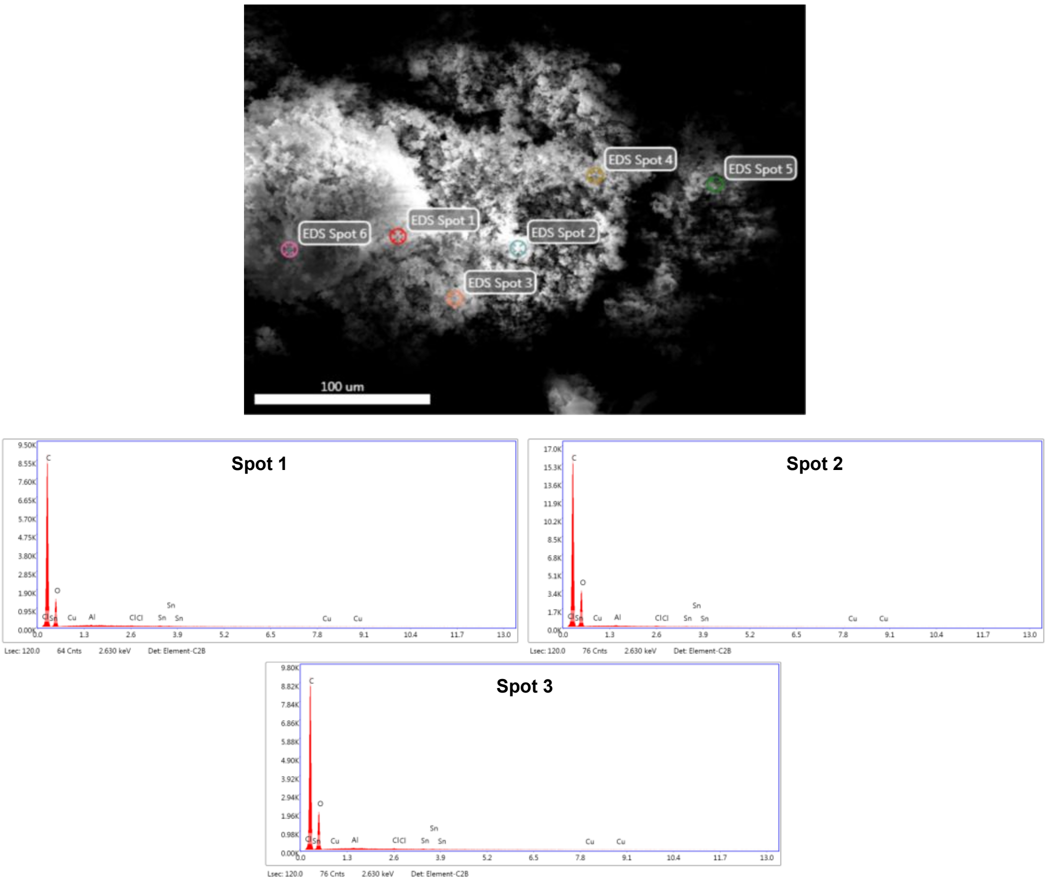

Figure S11. *SEM-EDX analysis of the PVMA employed in antimicrobial tests indicating negligible presence of metal traces.*
